# Public Health Aspects of Paediatric Dental Treatment under General Anaesthetic

**DOI:** 10.3390/dj4020020

**Published:** 2016-06-08

**Authors:** William Murray Thomson

**Affiliations:** Sir John Walsh Research Institute, Department of Oral Sciences, School of Dentistry, The University of Otago, Dunedin 9054, New Zealand; murray.thomson@otago.ac.nz; Tel.: +64212797116

**Keywords:** anesthesia, dental, child, preschool, quality of life

## Abstract

Early childhood caries (ECC) has negative psychosocial effects on children, with chronic pain, changed eating habits, disrupted sleep and altered growth very common, and it disrupts the day-to-day lives of their families. The treatment of young children with ECC places a considerable burden on health systems, with a considerable amount having to be provided under general anaesthesia (GA), which is resource-intensive. Justifying its use requires evidence of the efficacy of treatment in improving the lives of affected children and their families. This paper discusses the available evidence and then makes some suggestions for a research agenda.

## 1. Introduction

Despite improvements in dental caries rates over recent decades, early childhood caries (ECC) remains a highly prevalent, chronic disease among young children [1]. It places a considerable burden on health systems; in New Zealand, for example, dental treatment under general anaesthesia (DGA) is provided to approximately 5000 New Zealand children each year [2], with disproportionate numbers of Māori and Pacific Island children undergoing such treatment, along with those residing in deprived areas [3].

Dental caries is a multifactorial disease with causes and influences at a number of levels [4]. In fact, as with other chronic noncommunicable diseases, early childhood caries meets the criteria for designation as a “wicked” health promotion problem [5]: it is difficult to solve because of its complex, multi-level causes, and there is no single, universal solution. Preventive efforts are imperfect: relying solely on people’s personal behaviour is inadequate, because to be effective, self-care—such as sustained, long-term plaque control [6] and the use of self-applied topical fluorides (such as fluoride toothpaste)—needs to be done well and be carried out over the long term. The effectiveness of clinical preventive efforts (those administered by dental practitioners) for the group at highest ECC risk remains unclear at this stage. At community level, water fluoridation is effective [7], but it does not eliminate the disease; rather, it shifts the population disease distribution, and the problem of its long “tail” and the numbers requiring DGA remain [8]. Not only is sugar intake the most important person-level risk factor for the disease [9], the marketing and consumption of sugars is increasing [10] as part of the energy-dense and nutritionally compromised industrial diet—highly processed and convenient “junk” food—which has become more and more common, especially in the groups of low socio-economic status. Such food is high in sugar (much of which comes from high-fructose corn syrup), salt and fat, and it has low nutritional value. Being cheap, readily available and requiring minimal preparation, it tends to be consumed by those on low and/or insecure incomes [11], among whom the caries burden is greatest. 

There are strong socio-economic gradients in ECC occurrence, and it is likely that, as societal inequality continues to increase under the neoliberal hegemony [11], the incidence of the disease will continue to rise, leading to further increases in the numbers of children requiring dental treatment under GA. The bulk of those cases will be residing in deprived households, and so it is unlikely that there will be a fall in the numbers of children requiring DGA in the medium term.

## 2. The Effects of ECC on Children and Their Families

ECC has been shown to have negative psychosocial effects on children and to disrupt the day-to-day lives of their families. These effects are worsened by time spent on waiting lists for treatment.

Chronic pain, changed eating habits, disrupted sleep and altered growth are very common [12]. Among children prior to being treated under GA, chronic pain has been reported for at least two-thirds of them; eating difficulties have been experienced by 42% to 71%, and sleep disruption has affected between one-quarter and one half [13,14,15], although a Turkish study reported that 95% had sleeping difficulties [16]. Anyone who has children will recognise that a child with sleeping difficulties also disrupts the sleep of at least one parent. Indeed, the data show that the effects on the wider family are considerable [17]. Perhaps the greatest evidence for the impact of ECC on children and their families is the improvement in oral-health-related quality of life (OHRQoL) scale scores which is observed after the condition has been treated. This issue is further explored later in this paper.

## 3. Treating Children under GA

The number of children requiring dental treatment under GA has been a problem in New Zealand since the 1980s [8], but the problem is not confined to that country. The intervention rate in Australia trebled over the two decades from 1993–94 [18], and similar observations were reported from England [19].

Providing dental treatment under GA is far more expensive than conventional care, given that it requires an operating theatre, dentist, dental assistant, anaesthetist, anaesthetic technician and a recovery nurse (and room), as well as the conventional dental operating equipment. Given the expense (At around NZD$2,400 per case; [20]) and the sheer volume of cases, dental GA lists are vulnerable to the scrutiny of health service managers, whose brief includes maintaining and improving (where possible) the system’s efficiency. By contrast, the clinician’s main concern is treating the problem of the patient in his/her dental surgery at the time; if that can be achieved less traumatically and more efficiently by treating a case of early childhood caries under GA, that will be the option chosen. Repeated fruitless attempts at treating a young child with too much disease are traumatic for patient and clinician alike. Some years ago, a manager in New Zealand’s health system suggested that the paediatric dental “logjam” in his service’s theatres could be virtually eliminated by halting all treatment for the deciduous dentition; after all, went his logic, they were only baby teeth and would be replaced in time anyway. Those of us working in the dental care system reacted with predictable horror: we were aware of the benefits for child and parent alike from such treatment, but we had no data with which to demonstrate that. At best, we had some routinely collected statistics on throughput and, in some services, information on the treatment provided was able to be gleaned from claims data. Curtailing the treatment of paediatric caries cases under GA would have adverse consequences for children, households and the school dental services (who would have to struggle with treating those cases conventionally). There was an urgent need to obtain data on the wider effects of the disease and its treatment upon children and their families, and on their oral-health-related quality of life (OHRQoL).

## 4. Effects of Dental Treatment under GA on OHRQoL 

OHRQoL has been defined as “a standard of the oral tissues which contributes to overall physical, psychological and social wellbeing by enabling individuals to eat, communicate and socialise without discomfort, embarrassment or distress and which enables them to fully participate in their chosen social roles” [21]. It seemed as if it should be relatively easy to determine the effects on children (and their families) of treatment under GA. The problem was that there was (as yet) no validated scale for use with preschool children and their parents. Early studies had used single-item approaches or batteries of items to quantify individual aspects of the improvements associated with treatment under GA [22,23,24,25], but these had not been psychometrically validated and did not allow determination of effect sizes.

What was needed was a way of placing sufferers (whether children or their families) accurately on what would effectively be a “continuum of misery” and then observing their treatment-associated movement towards the less severe end of such a continuum. Fortunately, it was not long before the means to do so was available, with the emergence of the Parental-Caregivers Perceptions Questionnaire (P-CPQ), the first OHRQoL scale developed for use with very young children [26]. A separate Family Impact Scale (FIS) was developed for use alongside it. The higher the scale score, the more severe the impact. The scales are intended for use with younger children and their families. The 33-item P-CPQ has four subscales (oral symptoms, functional limitation, emotional well-being and social well-being), and the 14-item FIS has three (parental emotions, parental/family activity, and family conflict).

Short-form versions have been developed more recently in order to lessen the respondent burden. This process has resulted in two closely related sets of measures: the 13-item Early Childhood Oral Health Impact Scale (ECOHIS) [27]; and the short-form P-CPQ (with 8- and 16-item versions available) and 8-item FIS [28]. Both measures arose from the pioneering work of Jokovic and Locker, but differ in how they were developed [29]. In short, the ECOHIS was developed using an epidemiological sample, and the short-form P-CPQ and FIS measures arose from secondary analysis of data from two New Zealand studies of OHRQoL changes in ECC-affected children undergoing dental treatment under GA. Unsurprisingly, perhaps, a direct comparison of the properties and responsiveness of the measures found that the ECOHIS was less suitable for investigating DGA-treatment-associated changes in OHRQoL in young children, but the findings suggested that it might be more suitable for epidemiological use [29].

Such scales have been used extensively in observing treatment-associated changes in OHRQoL (Table 1). The design used in those studies has usually been a pre-/post-intervention one, with the same children assessed before treatment and again some time after it. Ethical and practical challenges have meant that only one [30] used a concurrent control group which did not receive treatment. The children in that control group were eventually treated, however. To date, 10 such studies have been published [13,14,15,16,30,31,32,33,34,35], with patient numbers at baseline ranging from 31 to 311, and follow-up rates ranging from 64% to 100% (with the latter seen in half of the studies). Of the five studies with less-than-perfect follow-up rates, three included attrition analyses, and none reported any statistically significant (or otherwise important) differences between those assessed at follow-up and those who were lost.

Examining before- and after-treatment scores on OHRQoL scales can be done in two main ways. The first involves comparing mean scale (and subscale) scores before and after treatment, with the computation of effect sizes. The effect size is a unitless measure of change which is calculated by dividing the mean change score (the difference between the baseline and follow-up score) by the standard deviation of the baseline scores, in order to give a dimensionless measure of effect. Effect size statistics of less than 0.2 indicate a “small” clinically meaningful magnitude of change, 0.2 to 0.7 a “moderate” change, and more than 0.7 a “large” change [13]. Effect sizes reported to date are presented in Table 2. Most are moderate or large, and there is little difference between the different scales which were used.

The second approach is to determine the prevalence of one or more impacts (say, “fairly often” or “very often”) at baseline and follow-up, and then to report the treatment-associated fall in prevalence. The latter does not use the full range of observed scores (and their variance) in the sample, but it has the distinct advantage of being easier to explain to lay people (including the all-important policy-makers and health service managers who control the resources for dental care). Data on DGA-associated changes in impact prevalence have not yet been reported in the literature. 

Data on changes in the prevalence of impacts do indeed make interesting reading. In the Lithuanian sample [15], the prevalence of any impact determined by the ECOHIS-Child and ECOHIS-Family fell from 98.4% and 95.9% (respectively) at baseline to 73.8% and 46.7% (respectively) at follow-up [36]. Reanalysis of the data from the Wellington (New Zealand) sample [13] showed that the prevalence of any impact determined by the P-CPQ fell from 96.3% to 67.5% (for one or more impacts reported “Sometimes”, “Often” or “Every day or almost every day”), and from 68.8% to 26.3% (for one or more impacts reported “Often” or “Every day or almost every day”). For the Auckland (New Zealand) sample [14], the prevalence of any impact determined by the P-CPQ fell from 95.5% to 75.5% (for one or more impacts reported “Sometimes”, “Often” or “Every day or almost every day”), and from 60.0% to 29.1% (for one or more impacts reported “Often” or “Every day or almost every day”). In other words, impact prevalence fell from about two-thirds to just over one-quarter of the children treated. Where the FIS was concerned, impact prevalence in the Wellington and Auckland samples fell from 82.5% and 67.3% to 33.8% and 44.5% (respectively) for one or more impacts reported “Sometimes”, “Often” or “Every day or almost every day”. For one or more impacts reported “Often” or “Every day or almost every day”, those proportions fell from 50.0% and 35.5% to 12.5% and 10.9% (respectively). As previously mentioned, falls in impact prevalence are likely to be a more effective “sell” to managers and policy-makers. Overall, the international data indicate that treating severe ECC cases under GA has benefits for both child and family.

## 5. Qualitative Investigations

Recent years have seen qualitative studies of the DGA experience appearing in the literature. These both complement and extend the quantitative reports. The first to appear was a study [37] in which Vancouver parents’ experiences of their child’s DGA were explored through interviews conducted at a postoperative follow-up appointment. The study’s aim was to shed light on the reasons for the DGA event not invariably being a spur to subsequent caries-promoting practices and behaviours in the affected household. Interestingly, one of the main themes to emerge was the role of the stresses of daily life in creating and sustaining barriers to caring for the child’s teeth. This raises the interesting possibility that children from more resilient and better-functioning families might not only have less disease but the effects of that disease on the family when it does occur might also be less disruptive. Guilt, stress and anxiety all featured in parental responses, as did affirmation of the positive benefits of the child having had the disease treated. 

In contrast to the parent-focused Canadian work, two recent reports by researchers at the University of Sheffield described their novel approaches to investigating the DGA experience from the child’s viewpoint. Video diaries were used alongside semi-structured interviews to obtain new insights into the process; these were not entirely negative, with greater attention and positive feedback from family members going some way to offsetting the more predictable anxiety, pre-operative hunger and discomfort [38]. The second report highlighted the need for greater involvement of the children in both the decision-making and the actual DGA process [39].

## 6. A Research Agenda—What Else Do We Need to Know?

There are a number of areas where more research is required (summarised in Table 3), and those can be considered under the three domains of the instrument, the care provided, and the household context (Table 3). Each will be discussed briefly. 

Concerning the instrument used, the first decision is which one to use. Of the studies to date, exactly half have used the ECOHIS and half have used the P-CPQ/FIS, and there were similar findings in terms of responsiveness and the effect sizes which were observed (although the ECOHIS-Family did show a substantially smaller effect size). A recent study directly compared the performance of the two measures in a secondary analysis of data from two pre-post-DGA studies conducted in New Zealand [29]. It found that, overall, the ECOHIS-Child and the P-CPQ scales are very similar in their internal consistency reliability, cross-sectional construct validity and responsiveness, at least for determining changes in OHRQoL associated with treatment for ECC. By contrast, the ECOHIS-Family and the FIS-8 differ in some important ways, despite being similar in their responsiveness. The former’s face validity is the most important weakness; of the three family impact domains (parental emotions, parental/family activity and family conflict) one is not sampled at all, and another is represented by a single item. In particular, the omission of the disrupted sleep item is particularly curious, given the high prevalence and impact of disrupted sleep for the parents of a child with ECC [29]. Accordingly, the P-CPQ and FIS scales are likely to be more suitable for use in DGA outcomes research.

Test-retest reliability remains an issue in this work. To date, there have been no investigations of this. Ethical concerns are likely to be the reason for this: requiring parents who are already undergoing a stressful time (as highlighted by the Canadian study [37]) would have been an undue imposition on them. Moreover, it would probably have affected follow-up rates. 

Other technical issues are those of (a) regression to the mean; (b) response shift; and (c) the sustainability of effects. Regression to the mean is always an issue with any examination of change scores, whereby some of the observed change arises from a phenomenon whereby those with more extreme baseline scores tend to have less extreme scores at follow-up, regardless of any real change in the characteristic being measured [40]. The Wellington study [13] examined this issue and adjusted the change scores by the mean change score for children for whom OHRQoL since the operation was judged (by parents using the global measure) to be “the same”. To do this, the mean baseline scores were corrected by that amount and then the effect sizes were recalculated. This did attenuate the effect sizes somewhat, but they were still substantial and clinically important, meaning that the OHRQoL of children undergoing DGA does improve measurably. Response shift is a phenomenon whereby individuals’ internal standards, values and view of their quality of life change as they adapt to their new situation [41]. To date, the nature, timing and extent of any response shift in respect of DGA and OHRQoL remain unclear, but it is likely to be detectable. This then raises the issue of the sustainability of the effects of DGA on OHRQoL: how long do they last, given the likelihood of response shift recalibrating informants’ perspectives? To date, this has not been reported, although Jankauskiene and co-workers are currently investigating it [36]. 

The nature of the treatment rendered under DGA is another important aspect. The two alternative approaches to treatment provision in such situations are: (1) the restorative/rehabilitative one (where every effort is made to restore diseased teeth, and extraction is undertaken only as a last resort); and (2) the exodontic one (where affected teeth are usually extracted and little or no effort is expended on saving teeth). It might be expected that the two would differ in terms of their effects on child and family OHRQoL, but that has not yet been reported in the literature. Similarly, their respective longer-term effects on the occlusion remain unknown because of a lack of good-quality longitudinal data.

Parents’ reports of their child’s OHRQoL and its effects on the family are likely to be influenced by characteristics such as personality and family functioning. Personality is known to influence people’s self-reported oral health, with those scoring higher on negative emotionality (or neuroticism) likely to report more negative impacts, other factors being equal [42]. What remains currently unclear is the influence of family functioning on the changes in OHRQoL: that is, whether the effects of the child’s condition on the family differ according to how well (or conversely how badly) the family (household) operates from day to day anyway. Domains of interest include problem-solving, communication, roles, affective responsiveness and involvement, and behaviour control, all of which can be captured in an instrument such as the McMaster Family Assessment Device [43]. All could be considered to have some effect on responses to the ECOHIS-Family or the FIS. To date, that has not been investigated, but it is likely that those from families which are more dysfunctional or chaotic will report less favourable outcomes from the DGA experience. It is surely only a matter of time before an expansion of knowledge in this area. There are also likely to be cross-cultural differences in ECC impact and in the responsiveness of the measures used; to date, these have not been explored. However, it is worth noting that the P-CPQ, FIS and ECOHIS were all developed from a common item pool which was generated from a wide variety of cultures, and so this may not be as important a consideration as might be expected.

## Figures and Tables

**Table 1:** Overview of studies of changes in child oral-health-related quality of life (OHRQoL) scale scores following dental treatment under GA.

Study	Country	Number at Baseline	Number at Follow-Up	Scale Used
Malden *et al.* [13]	New Zealand	202	130 (64%)	P-CPQ and FIS (full scales) ^a^
Klaasen *et al.* [31]	The Netherlands	31	31 (100%)	P-CPQ and FIS (full scales) ^b^
Klaasen *et al.* [30]	The Netherlands	144	104 (72%)	ECOHIS
Lee *et al.* [32]	Hong Kong	32	32 (100%)	ECOHIS
Gaynor *et al.* [14]	New Zealand	157	144 (92%)	P-CPQ and FIS (full scales) ^a^
Baghdadi *et al.* [33]	Saudi Arabia	67	67 (100%)	P-CPQ and FIS (short-form version)
Jankauskiene *et al.* [15]	Lithuania	140	122 (87%)	ECOHIS
Cantekin *et al.* [16]	Turkey	311	311 (100%)	ECOHIS
Almaz [34]	Turkey	120	98 (82%)	ECOHIS
Ridell *et al.* [35]	Sweden	75	75 (100%)	P-CPQ and FIS (full scales)

^a^ Data were subsequently reanalysed by Thomson *et al.* [28] using the short-form versions. ^b^ Scale data were not reported appropriately in this study.

**Table 2:** Treatment-associated effect sizes detected, by scale.

Study	Scale Which Was Used			
	P-CPQ	FIS	ECOHIS-Child	ECOHIS-Family
Malden *et al.* [13]	0.9	0.8	—	—
Klaasen *et al.* [30] ^a^	—	—	0.9	—
Lee *et al.* [32]	—	—	0.6	0.8
Gaynor *et al.* [14]	0.9	0.5	—	—
Baghdadi *et al.* [33]	1.6	1.5	—	—
Jankauskiene *et al.* [15]	—	—	1.6	2.4
Cantekin *et al.* [16]	—	—	0.9	1.3
Almaz [34]	—	—	1.0	1.2
Ridell *et al.* [35]	0.7	0.7	—	—
Mean effect size detected	1.0	0.9	1.0	1.4
Thomson *et al.* [29] ^b^	1.2	0.9	0.9	0.6

^a^ Effect sizes were unable to be calculated from the study data of Klaasen *et al.* ^b^ This was a secondary analysis of pooled data from the Malden et al and Gaynor et al studies; it calculated short-form scores for the P-CPQ and FIS, allowing direct comparison with ECOHIS scale scores in the same cohort.

**Table 3:** Areas requiring more research in the dental treatment under general anaesthesia (DGA) field.

Domain	Issues for investigation
The instruments	ECOHIS vs P-CPQ/FIS
	Test-retest reliability
	Reference period
	Response shift, sustainability of effects
	Regression to the mean
The care provided	Restorative/rehabilitative *versus* exodontic
	Longer-term orthodontic and other effects
The context	Roles of family function, parental personality
	The nature and extent of any cross-cultural differences
